# Olfactory and Cognitive Performance Improvement After Oxygen–Ozone Major Autohemotherapy in Mild Cognitive Impairment: A Retrospective Cohort Study

**DOI:** 10.3390/neurolint18030041

**Published:** 2026-02-24

**Authors:** Alessandro Micarelli, Simona Mrakic-Sposta, Sandro Malacrida, Alessandra Vezzoli, Riccardo Xavier Micarelli, Beatrice Micarelli, Ivan Granito, Marco Alessandrini

**Affiliations:** 1 Unit of Neuroscience, Rehabilitation and Sensory Organs, UNITER ONLUS, 00153 Rome, Italybeatricemicarelli@hotmail.it (B.M.); granito.ivan@gmail.com (I.G.); 2 EPOCA HEALTH, 00153 Rome, Italy; 3 ALTAMEDICA, 00198 Rome, Italy; 4 Institute of Clinical Physiology, National Research Council (CNR), 20162 Milan, Italy; simomrakic@gmail.com (S.M.-S.); alessandra.vezzoli@cnr.it (A.V.); 5 Institute of Mountain Emergency Medicine, Eurac Research, 39100 Bolzano, Italy; sandro.malacrida@eurac.edu; 6 Ear-Nose-Throat Unit, Department of Clinical Sciences and Translational Medicine, University of Rome Tor Vergata, 00133 Rome, Italy; malessandrini63@gmail.com

**Keywords:** mild cognitive impairment, olfactory dysfunction, Sniffin’ Sticks, oxygen–ozone therapy, major autohemotherapy, chemosensory–cognitive coupling

## Abstract

**Background/Objectives**: Mild cognitive impairment (MCI) is accompanied by olfactory dysfunction, and few interventions target shared chemosensory–cognitive mechanisms. We retrospectively examined whether a 5-week oxygen–ozone major autohemotherapy (MAH) cycle is associated with coupled improvements in olfactory and cognitive performance in adults with MCI. **Methods**: We analyzed 81 individuals with MCI who completed 10 MAH sessions (twice weekly) and 93 matched healthy controls. In the MCI group, olfactory function was measured before and after MAH using Sniffin’ Sticks^®^ threshold–discrimination–identification (TDI) scores; global cognition was assessed with the Mini-Mental State Examination (MMSE) and Montreal Cognitive Assessment (MoCA). We evaluated between-group and pre–post changes and used Spearman correlations to assess olfactory–cognitive coupling. **Results**: At baseline, MCI participants showed lower TDI and MoCA scores than controls and more hyposmia/anosmia. Following MAH, the proportion of normosmic patients increased from 32.1% to 50.6%, with fewer anosmic cases. TDI scores improved but remained lower than in controls. MMSE scores were unchanged, whereas MoCA total scores increased, with domain-level gains and a significant improvement in Language Repetition. TDI gains were modestly correlated with MoCA total and selected domain changes. **Conclusions**: In this retrospective cohort, MAH was associated with partial restoration improvements of olfactory function and improved cognitive performance. Correlated olfactory–cognitive changes were observed within the treated MCI group; however, causal attribution to O_2_–O_3_ MAH cannot be established without randomized, double-blind, sham-controlled trials with coupled olfactory–cognitive gains consistent with a shared, potentially modifiable substrate. Prospective randomized trials are needed to confirm efficacy and clinical utility.

## 1. Introduction

Mild Cognitive Impairment (MCI) represents a clinically heterogeneous transition state between physiological aging and overt dementia, typified by objective decline—most often in episodic memory—without substantial loss of everyday autonomy [1,2]. Prevalence estimates reach 15–20% among adults ≥ 65 years and, critically, 10–15% of cases convert to Alzheimer’s disease (AD) each year, with amnestic subtypes showing the steepest trajectory [3,4]. Because pharmacological disease-modifying therapies remain elusive, identifying early, pathophysiologically anchored biomarkers is essential for timely intervention.

Olfactory dysfunction, especially impaired odor identification, is one of the earliest and most sensitive harbingers of neurodegeneration. Cross-sectional work by Djordjevic and colleagues showed that individuals with MCI display higher detection thresholds and poorer identification accuracy than age-matched controls, correlating with memory and Attention scores [5]. A subsequent meta-analysis encompassing 31 studies (n ≈ 1900) confirmed a moderate-to-large overall effect (Cohen’s *d* = −0.76) and singled out identification as the most affected domain [6]. Notably, olfactory decline often precedes awareness of cognitive impairment and correlates poorly with global screening tools such as the Mini-Mental State Examination (MMSE), underscoring its potential as an independent biomarker [7]. Neuropathological investigations reveal that tau tangles and amyloid plaques accumulate early in olfactory-related regions—including the olfactory bulb, entorhinal cortex and hippocampus—mirroring the anatomical spread of AD [8,9].

Despite robust diagnostic value, therapeutic options that target olfactory impairment in MCI are virtually absent. One emerging candidate is oxygen–ozone (O_2_–O_3_) therapy, a century-old modality in which a calibrated O_3_/O_2_ mixture is delivered systemically (Major Autohemotherapy, MAH) [10]. Controlled exposure induces mild oxidative eustress that activates cytoprotective pathways such as Nrf2–ARE while dampening NF-κB-driven inflammation [11]. Systemic O_2_–O_3_ has been shown to suppress peripheral release of IL-1β, IL-6 and TNF-α, key mediators implicated in neuroinflammatory cascades. At the cellular level, ozone up-regulates PPARγ/Nrf2 signaling, alleviates reactive oxygen species production and stabilizes mitochondrial membrane potential, ultimately limiting parthanatos-like cell death [12,13]. Animal models reinforce these mechanistic findings: intraperitoneal ozone reduced amyloid-β load and improved spatial memory in APP/PS1 mice, partly via modulation of AKT phosphorylation [14]. Beyond basic science, a double-blind study in Parkinson’s disease reported improved postural stability with systemic O_2_–O_3_, and NIRS studies of major ozonated autohemotherapy in neurological cohorts have shown enhanced cerebral oxygenation and oxidative metabolism [15,16], in line with preclinical data that ozone activates Nrf2-driven antioxidant responses and has been reported to show neuroprotective-like effects in experimental models of Alzheimer’s and Parkinson’s disease [17].

Collectively, these pleiotropic actions map onto multiple pathogenic nodes of MCI—oxidative stress, neuroinflammation, vascular dysregulation and mitochondrial failure [12]. Given that the olfactory system is richly vascularized, metabolically active and uniquely exposed to environmental stressors [18,19], it may be particularly amenable to systemic redox–vascular modulation proposed for ozone-based interventions, ozone-mediated redox re-calibration, and microcirculatory enhancement [17].

Yet, no study has prospectively examined whether systemic O_2_–O_3_ therapy is associated with stabilization or improvement of olfactory function in MCI, despite the biomarker’s predictive power for AD progression and its objective quantifiability through standardized instruments such as the University of Pennsylvania Smell Identification Test (UPSIT) and Sniffin’ Sticks. Addressing this gap is clinically pertinent: improvement or preservation of olfaction may co-occur with broader changes in brain health and could serve as a clinically meaningful correlate of cognitive trajectories; however, causal inference regarding disease modification would require randomized sham-controlled trials.

The present investigation therefore seeks to determine whether MAH produces measurable changes in odor identification accuracy among patients with MCI. Secondary objectives include exploring correlations between olfactory outcomes and cognitive/functional indices, and delineating biomarker dynamics that might mediate treatment response. By targeting convergent molecular pathways with a single, low-cost intervention, our study aims to open a novel translational avenue for early dementia care—moving from passive observation of olfactory decline to proactive modulation of the underlying neurobiology.

## 2. Materials and Methods

### 2.1. Participants and Inclusion/Exclusion Criteria

This was a retrospective study involving 96 subjects with Mild Cognitive Impairment (MCI) who were enrolled for a routine protocol of MAH between January 2022 and April 2025. An expert clinician blinded to study outcomes (BM) diagnosed Mild Cognitive Impairment MCI according to National Institute on Aging/Alzheimer’s Association (NIA–AA) criteria [20], ensuring absence of dementia per NIA–AA recommendations [21]. Inclusion criteria were: (1) clinical diagnosis of MCI; (2) preserved basic Activities of Daily Living (ADLs) and no significant impairment in Instrumental ADLs (IADLs); (3) fluency in Italian; and (4) ability to provide informed consent by the participant or legally authorized representative. Global cognitive screening included the Mini-Mental State Examination (MMSE) and the Montreal Cognitive Assessment (MoCA), with MoCA favored for detecting mild deficits [22,23]. Diagnosis relied on overall clinical assessment plus neuropsychological evidence of objective impairment relative to age/education-adjusted norms, rather than any fixed MMSE cut-off, in line with consensus/guideline statements on MCI [20,24]. We used a computer-assisted frequency-matching procedure to select 93 healthy controls from the institutional database, matched to cases on gender, age, educational level, and BMI. Selection was performed by an independent biostatistician, blinded to study outcomes, using random sampling within strata and without replacement. All the participants underwent a general clinical and ear–nose–throat (ENT) examination. Chemosensory perception disturbances related to previous COVID-19 infection were considered as pre-enrollment exclusion criteria. Current or recent smokers (<3 years of abstinence) and individuals affected by allergies, metabolic diseases, Glucose-6-Phostate Dehydrogenase Deficiency (G6PD)-Favism and history of ENT surgery were excluded. Individuals with endocrinological disorders (in particular uncontrolled hyperthyroidism) or suffering from chronic renal disease and other systemic or organ failure disorders including neuro-psychiatric and cardiovascular disorders, as evaluated by medical history, physical examination and routine blood tests, were further excluded. Conditions of vegetarian/vegan diet, history of olfactory disturbances, ongoing use of medication (except oral contraceptives) and drug/alcohol abuse were considered as exclusion criteria. Gastrointestinal/eating disturbances and surgery (also detection of Helicobacter pylori excluded by a C13 urea breath test, but not history of appendectomy) and history of gustatory and/or smelling disorders (i.e., dysgeusia, parosmia, phantosmia) were considered as further drop-out conditions. Pregnant and currently breastfeeding females were excluded [25].

Global cognition was screened in all the participants with the Mini-Mental State Examination (MMSE; total score only) and the Montreal Cognitive Assessment (MoCA) [26]. Domain-level analyses were conducted only for MoCA according to previous experiences [27] because its item structure and validation specifically target separable Visuospatial, Naming, Attention, Calculation, Language Repetition, Language Fluency, Abstraction, and Delayed Recall e Orientation processes relevant to mild deficits [22,27]. Both MMSE and MoCA were administered in MCI participants at baseline and re-administered after the intervention with major ozone therapy (major autohemotherapy with ozone) to allow pre–post comparison.

### 2.2. Olfactory Testing

After a 12 h fast, between 7:00 and 9:30 a.m. [28] all participants underwent olfactory function testing before and immediately after the major autohemotherapy with ozone protocol. Smell function was assessed by means of the commercially available Sniffin’ Sticks^®^ test battery (Sniffin’ Sticks; Burghart Instruments, Wedel, Germany), a well-recognized tool to evaluate olfactory performance in clinical and research context [29]. It includes subtests for Odor Threshold (OT), Odor Discrimination (OD) and Odor Identification (OI), which are associated with different aspects of olfactory processing along the neural stream from olfactory bulb to the olfactory cortex [30] and were operationalized following previous procedures [29,31]. An interval of 3 to 5 min was applied between each subtest [32]. In each test the sum of correct answers can range from 0 to 16. The sum of all three subtests results in the composite TDI-score and reflects general olfactory capacity which thus can range from 0 to 48, with higher scores depicting a greater functionality [33]. To adjust for well-known gender differences in olfactory abilities and the age range of our cohort [34], we then categorized olfactory performance as abnormal using the <25th percentile TDI value from age- and gender-adjusted normative data (cut-off values for females were 32.35, 33.5, 33.5, 32.5, 30.75, 29.13 and 25.5 and for males were 30.75, 32.75, 32.76, 30.44, 29.25, 28.5, 22.75, respectively, for 11–20, 21–30, 31–40, 41–50, 51–60, 61–70 and 71–80 years sub-groups) [35].

All the participants were Caucasian adults and were recruited from UNITER ONLUS, a regional institutional inter-disciplinary disorder clinic, after their enrolment in the local longitudinal cohort study and from the University Hospital of Rome ‘Tor Vergata’. All the participants gave their written informed consent. The study was performed in agreement of the Declaration of Helsinki and was approved by the Institutional Ethics Committee (Reference number RS 60/20, date of vote: 24 July 2020).

### 2.3. MAH Protocol

MAH was administered twice per week for a total of 10 sessions (5 weeks), in line with consensus/standardization documents and clinical practice recommendations that endorse ≥2 sessions/week for systemic ozone therapy cycles (SIOOT-based protocols [36]) and are commonly applied in older adults [37,38,39,40,41,42]. The procedure followed ISCO3 standards (Madrid Declaration and the SIOOT-based MAH standard operating procedures [36,42]). Briefly, 100 mL of venous blood were drawn into an ozone-compatible certified ozone-resistant bag (TECNO_3_, Tecnoline Srl, Modena, Italy) pre-loaded with citrate anticoagulant (ACD-A, ~7–10 mL per 100 mL blood); the sample was exposed to a corresponding volume of O_2_–O_3_ mixture at low–medium systemic concentrations (typically 10–30 µg/mL, adjusted within this range according to individual clinical response and tolerability, according to the ISCO3 guidelines [42]) (OZO2FUTURA, Alnitec Srl, Cremona, Italy), gently mixed with a blood collection scales/bag mixer (EO51P-B, Vasini Strumenti Srl, Ravenna, Italy), and re-infused via the same venous access over ~10–15 min [36,40,41,42]. In accordance with safety guidance, concentrations >60–78 µg/mL and volumes >200 mL per session were avoided, particularly in older patients, and ozone-resistant materials (butterfly 19–21 G, certified tubing/filters) were used [36,41,42]. All participants underwent routine safety screening, including G6PD deficiency (absolute contraindication) and clinical monitoring during/after each session [36,41,42]. The choice of a twice-weekly × 10 schedule was based on SIOOT-standardized practice and prior clinical applications reporting at least two weekly sessions of MAH per cycle, with titration within the 10–30 µg/mL range according to tolerance and clinical context [10,36,38,41,42].

### 2.4. Data Handling and Statistical Analysis

Following previous experiences strongly associating olfactory dysfunction with MCI [43], the sample size was calculated to detect inter-group differences in the results for TDI. The total sample size was estimated to detect a mean difference between groups of 16% reduction in mean TDI, with a two-sample *t*-test and a significance level of α = 0.05 and a power of at least 80%. The estimated control group mean (SD) TDI of 30.2 (1.1) used gender- and age-balanced published data [43]. The choice to use the TDI composite score—rather than their sub-items—as main outcome measures is supported by the literature [25,44,45], and it is related to their main clinical significance in diagnosis and follow-up of olfactory performance [33]. The sample was further enlarged due to an estimated dropout associated with COVID-19 outbreak which—burdening the olfactory perception of patients—could have further dropped patients out of the study [25]. The chi-squared test was carried out to define associations between categorical factors and groups. Given their quantitative nature, descriptive data were calculated as mean and SD for olfactory and cognitive data. In order to assess that data for independent samples were of Gaussian distribution, D’Agostino’s K-squared normality and Levene’s homoscedasticity test were applied (where the null hypothesis is that the data are normally and homogeneously distributed). A between-group analysis of variance was performed for each variable. Gender was treated as a categorical predictor, and age, BMI and educational level were treated, where possible, as continuous predictors. The significant cut-off level (α) was set at a *p*-value of 0.05. Bonferroni correction for multiple comparisons was used for the post hoc test of the significant main effects, and the corrected level of significance was set at 0.017. Effect sizes for within-patient pre–post changes were computed as Cohen’s *d* using the pooled standard deviation of pre- and post-intervention scores. Within the MCI cohort, we computed two-tailed Spearman’s rank correlations among pre–post change scores (Δ = Post − Pre) across variables, using pairwise-complete observations (available-case analysis) and socio-demographic variables. Spearman’s ρ was selected as a nonparametric measure of monotonic association and is robust to non-Gaussian distributions and outliers [46,47]. For each family of tests (i.e., all pairwise Δ–Δ correlations assessed in the MCI group), *p*-values were adjusted using the Benjamini–Hochberg False Discovery Rate (FDR) procedure with *q* < 0.05 denoting statistical significance [48]. The magnitude of ρ was interpreted as: negligible (|ρ| < 0.10), weak (0.10–0.39), moderate (0.40–0.69), strong (0.70–0.89), and very strong (≥0.90) [49]. As an additional exploratory analysis, we computed age-stratified (<65 vs. ≥65 years) two-tailed Spearman correlations between baseline olfactory performance (TDI and OI) and baseline global cognition (MoCA) within the MCI cohort; we also compared baseline MoCA distributions between anosmic and non-anosmic participants within each age stratum. Analyses were performed in STATISTICA v7 (StatSoft Inc., Tulsa, OK, USA) for computation and GraphPad Prism v6 (GraphPad Software, San Diego, CA, USA) for visualization.

## 3. Results

### 3.1. Sample Characteristics and Participant Flow

**Figure 1:**
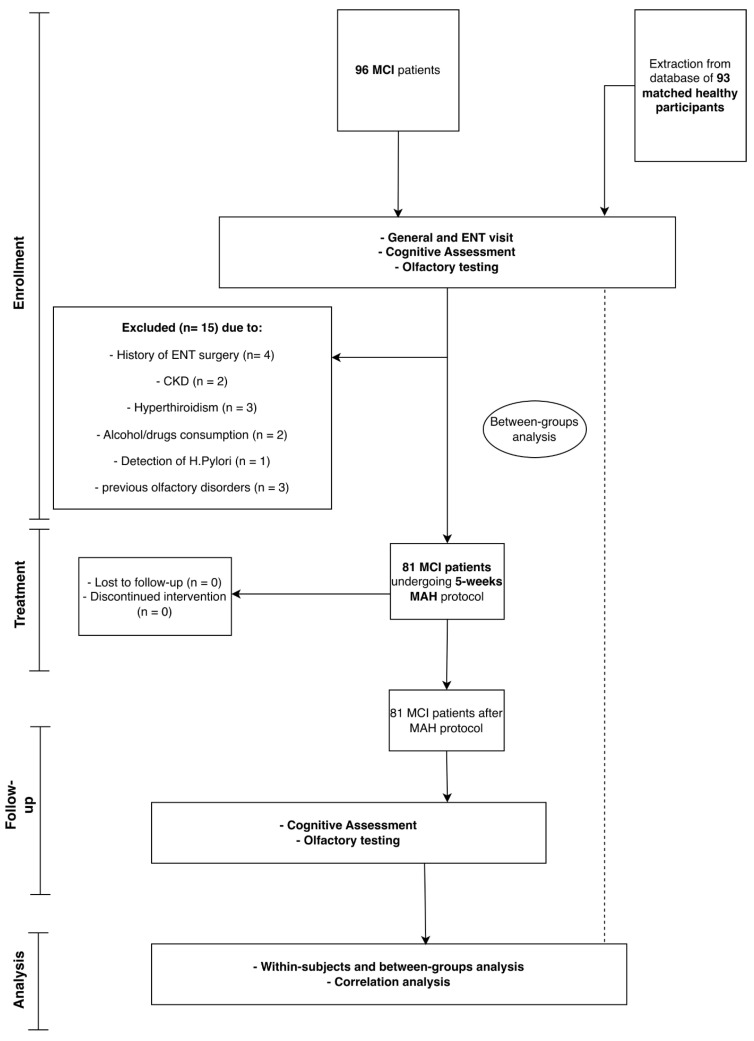
Flow diagram of participant enrollment, allocation, and analysis. A total of 96 consecutive patients with mild cognitive impairment (MCI) were screened. Fifteen patients were excluded due to predefined criteria (history of ear-nose-throat [ENT] surgery, chronic kidney disease [CKD], hyperthyroidism, alcohol/drug consumption, detection of Helicobacter pylori, or previous olfactory disorders), leaving 81 eligible MCI participants who underwent baseline general and ENT evaluation, cognitive assessment, and olfactory testing, and subsequently completed the 5-week major autohemotherapy (MAH) protocol without loss to follow-up or treatment discontinuation. Ninety-three matched cognitively healthy individuals were extracted from an institutional database and included as controls for between-groups analyses. Within-subject pre–post comparisons, between-groups analyses, and correlation analyses were conducted on the subsets indicated in the diagram.

A total of 96 consecutive patients with a clinical diagnosis of MCI were screened for eligibility (Figure 1). Fifteen individuals were excluded due to major medical comorbidities, previous otorhinolaryngological surgery or other predefined criteria, leaving 81 MCI participants who completed the full 5-week MAH protocol without loss to follow-up or treatment discontinuation (Figure 1).

The control group consisted of 93 cognitively healthy individuals frequency-matched for age, sex, education and BMI. As summarized in Table 1, there were no significant between-group differences in gender distribution, age, years of education or BMI (all *p* > 0.05), indicating adequate baseline comparability on key socio-demographic variables.

### 3.2. Distribution of Olfactory Categories Before and After MAH

**Figure 2:**
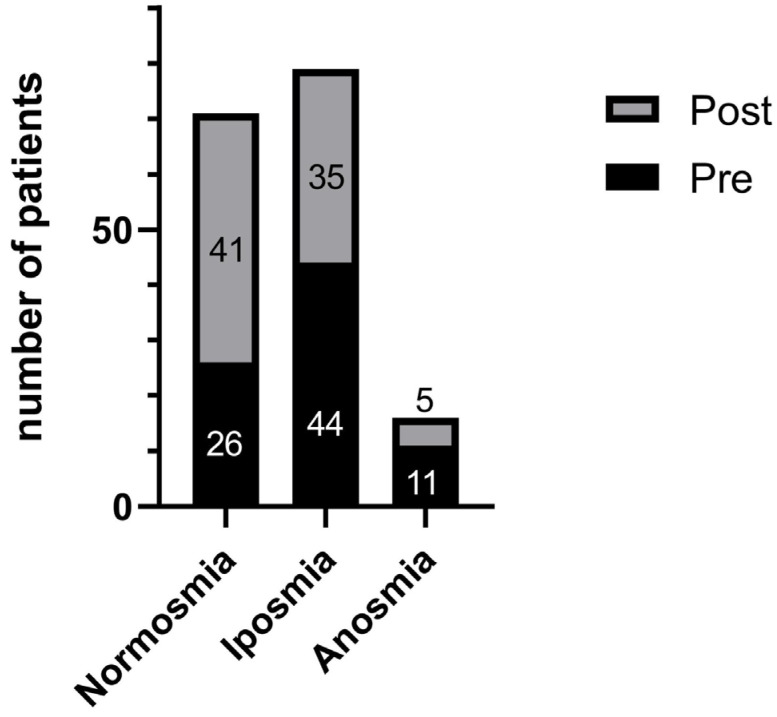
Normosmic and dysosmic individuals before and after the MAH protocol. Stacked bars depict the number of MCI patients classified as normosmic, hyposmic, or anosmic according to age- and sex-adjusted Sniffin’ Sticks TDI cut-offs at baseline (Pre) and after completion of the MAH cycle (Post). At baseline, 26 patients were normosmic, 44 hyposmic, and 11 anosmic; after MAH, 41 were normosmic, 35 hyposmic, and 5 anosmic, indicating a redistribution toward normosmia. Group differences in category distribution were evaluated using chi-square tests (see text).

At baseline, only 26/81 (32.1%) MCI patients were normosmic, whereas 44/81 (54.3%) were hyposmic and 11/81 (13.6%) anosmic according to age- and sex-adjusted TDI cut-offs. Following the MAH cycle, the proportion of normosmic individuals increased to 41/81 (50.6%), while hyposmics decreased to 35/81 (43.2%) and anosmics to 5/81 (6.2%) (Figure 2). The shift in olfactory-category distribution from pre- to post-intervention was statistically significant (χ^2^ = 6.63, *p* = 0.036), supporting a clinically meaningful improvement in chemosensory status in the treated MCI (Figure 2).

### 3.3. Olfactory and Cognitive Changes in MCI Patients and Comparison with Controls

**Figure 3:**
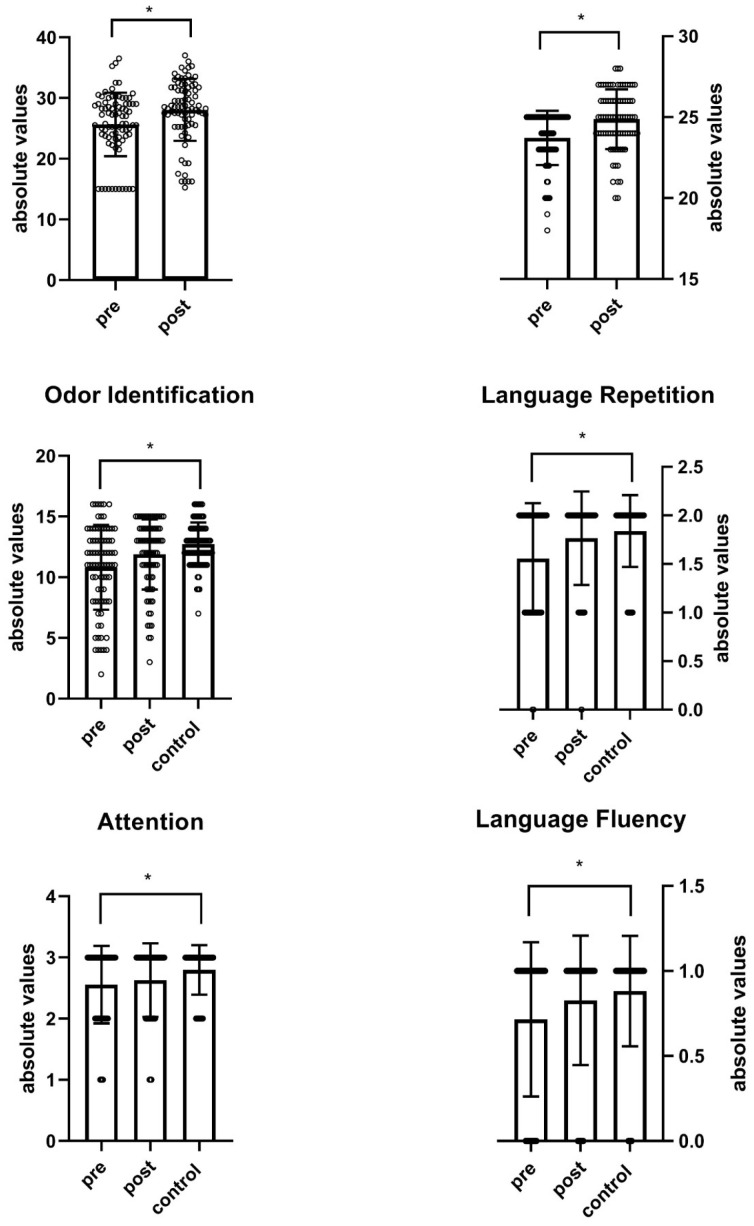
Significant changes in olfactory and cognitive performance. Bar plots (with individual data points) illustrate pre- and post-MAH values in the mild cognitive impairment group, and corresponding values in healthy controls where available, for TDI composite score, MoCA total score, Odor Identification, Language Repetition, Attention, and Language Fluency. Bars represent mean absolute scores; error bars indicate variability (standard deviation). Asterisks denote statistically significant differences (*p* < 0.05) based on omnibus ANOVA across MCI-pre, MCI-post, and control groups with appropriate post hoc comparisons, and/or paired pre–post tests within the MCI group (details in the main text). Abbreviations: TDI, Threshold–Discrimination–Identification; MoCA, Montreal Cognitive Assessment.

Detailed pre–post and between-group data are reported in Table 2 and illustrated in Figure 3.

Within the MCI group, MAH was associated with consistent improvements in quantitative olfactory performance. Although not significantly, mean OT increased from 4.84 ± 2.72 to 5.77 ± 2.85 (*p* = 0.036), OD from 9.99 ± 1.15 to 10.40 ± 1.36 (*p* = 0.04), and OI from 10.81 ± 3.49 to 11.89 ± 2.89 (*p* = 0.034), yielding small effect sizes (Cohen’s *d* ≈ 0.33–0.34). The composite TDI score increased from 25.65 ± 5.24 to 28.05 ± 5.11 (*d* = 0.46), indicating a significant (*p* = 0.035) moderate within-patient effect on global olfactory function. Despite these gains, post-MAH TDI values in MCI participants remained significantly (*p* < 0.001) lower than those of matched controls (31.34 ± 2.74), documenting partial but not complete normalization. On the other side, post-MAH OI values were found to be not significantly (*p* = 0.02) different when compared to control participants.

Cognitively, MMSE scores showed only a negligible pre–post change (24.46 ± 1.96 to 24.58 ± 1.97, *d* = 0.06, *p* = 0.69), whereas MoCA total scores improved from 23.72 ± 1.68 to 24.88 ± 1.84 (*d* = 0.66), consistent with a significant (*p* < 0.001) moderate enhancement in global cognitive efficiency after MAH. Beyond Language Repetition which was found to significantly (*d* = 0.40, *p* = 0.012) improve after MAH, all other MoCA subdomains exhibited small-to-moderate improvements, including Visuospatial abilities (*d* = 0.14, *p* = 0.39), Calculation (*d* = 0.17, *p* = 0.29), Abstraction (*d* = 0.37, *p* = 0.027), Delayed Recall (*d* = 0.26, *p* = 0.09), Orientation (*d* = 0.15, *p* = 0.3) and, to a lesser extent, Attention (*p* = 0.44) and Language Fluency (*p* = 0.09) (*d* ≈ 0.11). When compared to control participants post-MAH Abstraction, Attention, Language Repetition and Language Fluency scores were found to be not significantly different (*p* = 0.024, *p* = 0.03, *p* = 0.25 and *p* = 0.3, respectively). All the other MoCA subdomains remained significantly (*p* < 0.001) lower with respect to the control participants. Exploratory analyses of sex-by-time interactions suggested a small advantage for men in Orientation gains (F(1, 246) = 3.89, *p* = 0.04, mean ± SD = 5.42 ± 0.65 and 5.57 ± 0.54 for pre- and post-MAH in men, respectively, *d* = 0.25; mean ± SD = 5.66 ± 0.58 and 5.69 ± 0.62 for pre- and post-MAH in women, respectively, *d* = 0.05) and relatively higher Delayed Recall improvement compared with men (F(1, 246) = 4.79, *p* = 0.029, mean ± SD = 2.22 ± 1.24 and 2.48 ± 1.25 for pre- and post-MAH in men, respectively, *d* = 0.21; mean ± SD = 1.75 ± 1.22 and 2.13 ± 1.07 for pre- and post-MAH in women, respectively, *d* = 0.33); however, these effects were modest and should be interpreted cautiously.

### 3.4. Correlation Structure of Pre–Post Changes

**Figure 4:**
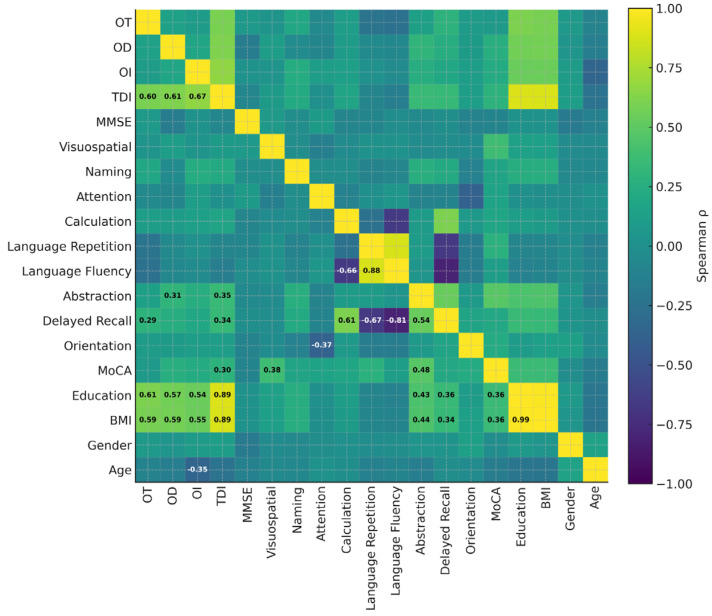
Heatmap displaying Spearman’s ρ computed on pre–post change scores (Δ = Post − Pre) for olfactory and cognitive variables and their associations with baseline socio-demographic indices. Cells depict Spearman’s ρ (warmer colors = more positive associations; cooler colors = more negative associations), with the main diagonal (ρ = 1) shown for completeness. Numeric values are displayed in the lower triangle only for associations surviving Benjamini–Hochberg false discovery rate correction (*q* < 0.05, two-tailed). Text color is adapted (light on dark cells, dark on light cells) to optimize readability. MoCA, Montreal Cognitive Assessment; MMSE, Mini-Mental State Examination; OT, Odor Threshold; OD, Odor Discrimination; OI, Odor Identification; TDI, Threshold–Discrimination–Identification composite score; BMI, body mass index (kg/m^2^). Age and education are expressed in years.

The multivariate pattern of associations between pre–post changes in olfactory and cognitive measures and baseline socio-demographic indices is summarized in the FDR-corrected Spearman correlation matrix (Figure 4).

As expected, improvements in TDI were strongly and positively correlated with changes in its constituent subtests (OT, OD, OI; all *q* < 0.001). Beyond this internal coherence, greater gains in global olfactory function were weakly-to-moderately associated with concurrent improvements in MoCA total scores and in specific higher-order domains, including Abstraction and Delayed Recall (*q* < 0.05), supporting a coupled enhancement of chemosensory and cognitive performance within the treated MCI cohort. Changes in selected cognitive indices also showed structured interdependencies, with robust correlations among language-related measures and between executive–memory subdomains, delineating a coherent cognitive response profile to MAH. Socio-demographic variables contributed to this network: higher educational attainment showed positive associations with several favorable Δ-scores, whereas age exerted a negative association with improvement in odor identification (*q* < 0.05). Associations involving BMI and education were interpreted with caution due to their collinearity in this sample. Overall, the correlation topology indicates that patients who benefited more strongly in olfactory function tended also to exhibit greater cognitive gains, in line with the hypothesized shared susceptibility of olfactory and frontal–temporal networks to redox-modulating interventions. Additionally, we performed an exploratory age-stratified analysis (<65 vs. ≥65 years) to test whether baseline olfactory performance was associated with baseline global cognition within the MCI cohort. Baseline TDI was not significantly correlated with baseline MoCA in either age stratum (Spearman’s ρ = 0.09, *p* = 0.614 for <65; ρ = −0.05, *p* = 0.734 for ≥65), and no significant association was observed also in the overall MCI sample (ρ = −0.01, *p* = 0.954).

## 4. Discussion

This retrospective study provides preliminary evidence that a standardized 5-week cycle of MAH is associated with measurable improvements in both olfactory and cognitive performance in older adults with MCI, in a design that does not allow causal inference (Table 2, Figure 3). At baseline, our MCI cohort exhibited expected quantitative olfactory deficits relative to matched healthy participants, with composite Sniffin’ Sticks TDI scores predominantly in the hyposmic range. Following MAH, we observed (i) a clinically meaningful increase in global olfactory function with redistribution of patients from hyposmia/anosmia toward normosmia (Figure 2), and (ii) a parallel, domain-selective enhancement in Montreal Cognitive Assessment (MoCA) performance (Table 2, Figure 3). The structured pattern of correlations between pre–post changes in olfactory indices and MoCA subscores is consistent with shared pathophysiological links between chemosensory function and higher-order cognitive performance in MCI; however, these associations are hypothesis-generating and do not establish causality. 

The structured pattern of correlations between pre–post changes in olfactory indices and MoCA subscores further suggests a shared, potentially modifiable substrate linking chemosensory and higher-order cognitive functions in MCI (Figure 4).

### 4.1. Olfactory Plasticity and Response to Oxygen–Ozone Therapy

A critical finding of this investigation is that olfactory dysfunction in MCI appears partially plastic and responsive to targeted redox-modulating therapy over the short term, showing pre–post changes in association with MAH. We documented a statistically significant and clinically meaningful increase in composite TDI scores (mean +2.40 ± 0.21 points, effect size *d* = 0.46, *p* = 0.035) (Table 2, Figure 3), accompanied by a substantial redistribution of participants toward normosmia: the proportion of normosmic individuals increased from 32.1% to 50.6%, while the proportion of anosmic patients decreased from 13.6% to 6.2% (χ^2^ = 6.63, *p* = 0.036) (Figure 2). The magnitude of change is particularly noteworthy given that Sniffin’ Sticks demonstrates has shown high test–retest reliability over similar timeframes, and all three TDI components (threshold, discrimination, identification) improved coherently, making pure measurement error less likely; nevertheless, practice/learning effects cannot be excluded in the absence of repeatedly tested or sham-treated controls rendering pure measurement error or learning effects unlikely as sole explanations [35,50].

Potential mechanistic interpretations of the observed pre–post associations should be regarded as hypothesis-generating, because no biological samples, physiological measures, or neuroimaging were collected in this cohort. In MAH, ozone is reacted ex vivo with autologous blood at calibrated concentrations, generating a short-lived redox signal; prior literature proposes that this can engage adaptive redox–immune pathways [51]. Ozone is a potent oxidant and, when inhaled, is a well-established pulmonary irritant, with controlled human exposure studies demonstrating reversible decrements in lung function and induction of airway inflammation in healthy subjects [52,53]. Therefore, any medical use of ozone requires strict precautions to prevent inhalational exposure and must be performed only with controlled, certified ozone generators and appropriate safety procedures [36]. Importantly, in MAH ozone is not administered by inhalation; rather, it is reacted ex vivo with autologous blood, thereby avoiding direct pulmonary exposure, although careful handling and adherence to standardized protocols remain essential [36]. 

In the context of our outcomes, improved olfactory performance could plausibly reflect reduced peripheral inflammatory signaling and downstream neuromodulatory normalization (including serotonergic mechanisms relevant to olfactory network function), but this remains speculative without biomarker confirmation [54,55]. More broadly, neurological applications of ozone therapy remain complex and incompletely defined, with limited high-quality evidence in CNS disorders [56]. The preclinical and heterogeneous clinical literature has proposed multi-system effects of ozone-related interventions; however, these mechanisms were not assessed here and should not be interpreted as demonstrated in this cohort [56].

The mechanistic basis for ozone-induced improvements in olfactory function is grounded in the hormetic oxidative eustress model. When administered as MAH at calibrated systemic concentrations (10–30 µg/mL), ozone is rapidly consumed in the ex vivo blood mixture, generating a transient pulse of reactive oxygen species and lipid ozonation products that triggers adaptive cellular responses rather than sustained damage. At the molecular level, this controlled oxidative stress activates the nuclear factor erythroid 2-related factor 2 (Nrf2)–antioxidant response element (ARE) axis, a master regulator of the cellular defense system. Nrf2 translocates to the nucleus, binds ARE sequences on DNA, and initiates transcription of phase II detoxifying enzymes and endogenous antioxidant proteins (heme oxygenase-1, NAD(P)H quinone oxidoreductase, glutathione-S-transferases) that provide sustained cytoprotection. Concurrently, ozone-induced Nrf2 activation and ERK1/2 phosphorylation suppress NF-κB-dependent inflammatory signaling, reducing production of pro-inflammatory cytokines (IL-1β, IL-6, TNF-α) implicated in neuroinflammatory cascades. These pathways may have impacted on the olfactory system, as the olfactory epithelium and bulb are—as stated—highly vascularized, metabolically active tissues with direct exposure to environmental toxicants and a recognized vulnerability to microvascular injury and neuroinflammation in aging and neurodegenerative disease.

Animal models provide complementary evidence for ozone-mediated neuroprotection. In APP/PS1 transgenic mice modeling familial Alzheimer’s pathology, systemic ozone therapy reduced APP protein levels, decreased amyloid-β accumulation, improved spatial and recognition memory, and restored hippocampal glutamate homeostasis, partly through modulation of AKT phosphorylation and synaptic density. Clinical evidence has also emerged: in acute ischemic stroke patients, adjunctive ozonated autohemotherapy was associated with better neurological recovery (30% reduction in NIHSS scores), reduced neuron-specific enolase and S100β, and up-regulation of Nrf2 signaling compared with oxygen-only controls. These findings suggest that ozone’s pleiotropic actions—including enhanced microcirculation, improved erythrocyte deformability and rheology, correction of endothelial dysfunction via nitric oxide pathways, and mitochondrial stabilization—may collectively restore or partially reverse the vascular–metabolic-inflammatory dysfunction that impairs olfactory and cognitive function in aging and MCI.

### 4.2. Coupled Enhancement of Olfactory and Cognitive Performance and Relationship to Olfactory Biomarkers

Beyond olfactory changes, MAH was associated with significant improvement in global MoCA scores (mean +1.16 ± 0.16 points, effect size *d* = 0.66, *p* < 0.001), contrasting sharply with negligible MMSE changes (*d* = 0.06, *p* = 0.69) (Table 2). This dissociation is consistent with the well-established superior sensitivity of the MoCA to early executive and episodic memory deficits characteristic of prodromal Alzheimer’s disease [22,57]. Domain-level MoCA analysis revealed selective enhancements in indices particularly vulnerable in early neurodegeneration. Delayed Recall (*d* = 0.26, *p* = 0.09) and Abstraction (*d* = 0.37, *p* = 0.027) showed non-significant numerical gains, whereas Language Repetition (*d* = 0.40, *p* = 0.012) remained significant after correction—implicating fronto-temporal and parietal networks. These targeted cognitive improvements parallel evidence that MoCA-derived memory indices (e.g., MoCA Memory Index Score) predict short-term conversion from MCI to AD dementia [58].

Most compellingly, correlation analyses revealed structured interdependencies between olfactory and cognitive improvements (Figure 4). Greater gains in global TDI, particularly odor identification, were weakly-to-moderately associated with concurrent enhancements in MoCA total scores and specific subdomains (Abstraction, Delayed Recall; all *q* < 0.05 after Benjamini–Hochberg false discovery rate correction). This coupling is neurobiologically plausible, grounded in the well-established role of olfactory dysfunction as an early biomarker of cognitive decline [59]. Recent works confirm that MCI patients show pronounced impairments in specific olfactory domains—especially odor identification and discrimination—with larger effect sizes in amnestic MCI subtypes, implicating a mechanistic connection between memory-related neurocircuits and olfactory networks [6,50,59]. Neuropathologically, the olfactory bulb and entorhinal cortex are among the earliest sites of tau and amyloid-β accumulation, preceding cortical pathology by years [9]. Recent reviews further reinforce olfactory dysfunction as an early clinical feature of neurodegeneration and as a readout of vulnerability along the olfactory–entorhinal–hippocampal axis [60,61]. Neuropathological and experimental evidence also links olfactory structures to Alzheimer’s disease biology: amyloid-β and tau pathology have been described in the olfactory epithelium in Alzheimer disease [62], and in transgenic models olfactory dysfunction correlates with amyloid-β burden [63]. In humans, odor-identification deficits have also been associated with amyloid burden assessed by PiB PET across Alzheimer’s disease, MCI and healthy aging cohorts [64].

With respect to mechanism, our dataset shows an observed clinical signal (pre–post improvements in olfaction and global cognition in association with MAH) but cannot discriminate causality between competing biological pathways. One plausible hypothesis is that attenuation of inflammatory signaling may mitigate neuromodulatory disruption relevant to olfactory bulb circuitry, including serotonergic dysregulation described in Alzheimer’s disease and implicated in olfactory network function [54,55]. In principle, improved serotonergic tone could facilitate more efficient information transfer along the olfactory–entorhinal axis; however, this remains speculative without direct neurochemical measures.

An alternative (non-exclusive) hypothesis is that systemic redox–immune modulation induced by MAH ozone therapy may extend beyond the olfactory periphery and influence broader neuroinflammatory signaling affecting limbic structures, including the entorhinal cortex. Mechanistically, medical ozone has been proposed to engage adaptive antioxidant and anti-inflammatory pathways (including Nrf2-related signaling) with downstream suppression of pro-inflammatory cascades [51,65,66]. This interpretation should be regarded as hypothesis-generating in the absence of central biomarkers or neuroimaging and should be tested in future trials incorporating AD-relevant biomarkers (e.g., plasma/CSF Aβ/tau, neuroinflammation markers) and structural/functional imaging.

As the olfactory system anatomically inputs directly to hippocampal, orbitofrontal, and limbic areas critical for episodic memory and executive functions [67,68], early pathology in these networks may disrupt both chemosensory processing and higher cognitive domains [69,70]. Both olfactory and fronto-temporal networks are vulnerable to shared pathological cascades including chronic neuroinflammation, endothelial dysfunction, impaired neurovascular coupling, mitochondrial failure, and tau pathology [71]. Therefore, systemic interventions targeting redox balance and vascular health may produce parallel benefits across these interconnected systems [72,73]. Our observation of coupled improvements in olfactory and cognitive functions following ozone-mediated redox and microvascular modulation supports the concept that chemosensory impairments in MCI—long recognized as early indicators of cognitive decline—may reflect dynamic, potentially interrelated pathophysiological processes affecting both chemosensory and cognitive function; causality and reversibility cannot be inferred from this design, but they could be at least partially reversible through correction of shared pathophysiology [12,74,75]. The lack of uniform improvements across all cognitive domains (e.g., minimal effects on Attention and Language Fluency) indicates specific, mechanism-driven effects rather than broad nonspecific changes.

Additional correlational analyses in our study highlighted nuanced and biologically plausible patterns of association beyond the primary olfactory and cognitive domains. Specifically, improvements in olfactory threshold and discrimination were positively correlated with gains in executive function and visuospatial abilities, suggesting that enhancements in sensory processing may parallel improvements in higher-order cognitive control and spatial orientation networks. This relationship is consistent with neuroanatomical evidence indicating shared connectivity between olfactory regions and prefrontal and parietal cortices, key substrates for executive and visuospatial processing [50,67,76]. Moreover, cognitive gains exhibited significant interdependence among memory, Abstraction, and language-related subdomains, reflecting the integrated functioning of frontotemporal and limbic networks that underpin complex cognitive operations and semantic processing [77]. Such clustered improvements suggest that the observed pre–post changes may involve network-level reorganization or increased functional coupling rather than isolated regional effects. Such clustered improvements suggest that redox and microvascular modulation by oxygen–ozone therapy may restore or enhance network-level interactions rather than isolated regional function.

Socio-demographic moderators also emerged as significant, with higher educational attainment positively correlating with greater cognitive and olfactory improvements. This aligns with cognitive reserve theory, which posits that education and related life experiences confer resilience against neurodegenerative processes by enhancing neural efficiency or compensatory mechanisms [78]. Specifically, cognitively enriched individuals may possess greater capacity for neural plasticity or alternate network recruitment in response to targeted interventions [79]. These findings imply that patients with greater cognitive reserve may benefit more from systemic interventions that target oxidative and vascular dysfunction, possibly due to a more plastic or responsive neural substrate.

**Figure 5:**
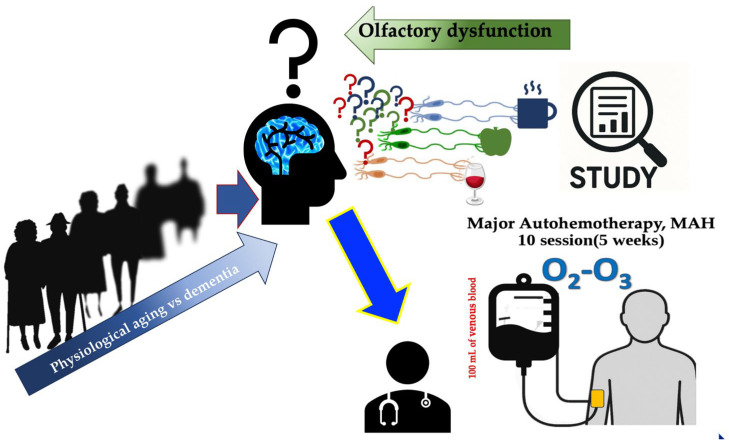
Conceptual hypothetical model of the study. Aging individuals with mild cognitive impairment (MCI) frequently present olfactory dysfunction, a clinical marker at the interface between physiological aging and dementia. In our cohort, patients with MCI and smell impairment underwent a 5-week cycle of 10 sessions of oxygen–ozone major autohemotherapy (MAH; 100 mL of autologous venous blood exposed to an O_2_–O_3_ gas mixture and reinfused under medical supervision). Olfactory function and global cognition were evaluated before and after the MAH cycle. The schematic summarizes the working hypothesis that oxygen–ozone-mediated modulation of systemic pathophysiology may lead to improved olfactory performance, enhanced cognitive function, and a potentially modifiable biological substrate in MCI. This schematic is a hypothesis-generating, hypothetical model integrating the observed pre–post changes with mechanisms proposed in the prior literature; no mechanistic biomarkers or neuroimaging were collected, and causal inference is not possible in this single-arm design.

Collectively, these structured interrelations reinforce the conceptualization of a coherent and dynamic biological network whose short-term changes were observed in association with oxygen–ozone MAH. Although no biomarkers were collected, the prior literature suggests that systemic redox–immune and vascular pathways may plausibly influence both chemosensory and cognitive domains; thus, any mechanistic interpretation remains hypothesis-generating and impacted by oxygen–ozone therapy. By modulating systemic oxidative stress, inflammation, and endothelial function, this intervention may exert widespread effects that unify chemosensory and cognitive pathways [12,13,56,80]. This view is supported by multimodal imaging studies demonstrating concurrent disruption and restoration functional reorganization of olfactory, executive, and memory-related networks in early neurodegeneration [74,81]. Thus, our correlative evidence is consistent with shared pathophysiological links between chemosensory and cognitive domains in MCI, but does not establish causality or therapeutic efficacy. Thus, our correlative evidence underscores the potential of targeting shared pathophysiological mechanisms to achieve broad-spectrum neural benefits in MCI (Figure 5).

### 4.3. Clinical Implications and Mechanistic Plausibility

Our findings carry important clinical implications. First, the pattern of MAH-associated improvements suggests that olfactory measures could serve not merely as static risk indicators but as sensitive intermediate outcomes in early-phase trials of interventions targeting inflammaging, microvascular dysfunction, or mitochondrial resilience. Second, the selective enhancement of MoCA and specific higher-order domains suggests potential utility of oxygen–ozone therapy as a non-pharmacological adjunct within multidomain interventions for MCI, together with physical exercise, cognitive training, vascular risk factor management, and dietary strategies—approaches now favored over cholinesterase inhibitors in current guidelines [24,82]. Regarding other anti-inflammatory strategies, non-steroidal anti-inflammatory drugs (NSAIDs) are widely used for peripheral inflammatory conditions; however, randomized and systematic evidence does not support NSAIDs for dementia prevention or as disease-modifying therapy in Alzheimer’s disease, and safety concerns (gastrointestinal, renal, and cardiovascular) are particularly relevant in older populations [83,84]. Third, they reinforce the emerging consensus that quantitative olfactory testing—interpreted with updated, age- and sex-stratified normative data—should be incorporated into routine cognitive screening for older adults with subjective cognitive concerns or objective mild deficits [6,50]. Recent longitudinal evidence suggests that the prognostic meaning of olfactory loss may be age-dependent: Kim and Cho [85] reported that anosmia diagnosed in middle age was associated with increased subsequent Alzheimer’s disease/dementia risk, whereas this association was not observed in older adulthood. While our short pre–post design cannot address incident dementia risk, an exploratory analysis within our MCI cohort did not show significant baseline associations between TDI and global cognition (MoCA) in either <65 or ≥65 years, which should be interpreted cautiously given the restricted cognitive range within established MCI and limited power for age-window effects. These considerations further support the need for prospective, adequately powered, age-stratified studies with long-term clinical and biomarker endpoints [85]. Standardized instruments such as Sniffin’ Sticks offer reproducible, low-cost biomarkers that may enhance diagnostic and prognostic precision when combined with MoCA and other cognitive measures.

### 4.4. Limitations and Future Directions

Despite these encouraging preliminary results, several methodological limitations warrant emphasis. The retrospective, single-center, single-arm study design precludes definitive causal inference. Regression to the mean, non-specific effects of clinical attention, and practice effects on Sniffin’ Sticks and MoCA cannot be fully excluded without sham-treated or repeatedly tested control arms and alternate test forms at follow-up. Practice effects are particularly relevant for MoCA, where repeated administrations have been associated with improved scores even in cognitively healthy older adults [86]. Sniffin’ Sticks components show good short-interval test–retest reliability [87,88]. Nevertheless, reliability does not exclude familiarity/learning effects, which cannot be quantified in the absence of alternate forms and control arms. Our follow-up captured only immediate post-treatment changes; the durability of olfactory and cognitive gains, their sustainability beyond the treatment cycle, and the potential impact on medium-term conversion rates to dementia remain unknown. Biological samples were not collected, preventing direct linkage between clinical improvements and specific molecular or vascular endpoints. Accordingly, any mechanistic interpretation in this manuscript should be regarded as hypothesis-generating rather than evidence-based. More broadly, the neurological application of ozone therapy remains complex and incompletely defined, with limited high-quality clinical evidence in central nervous system disorders [56]. Finally, despite careful exclusion of major confounders, residual confounding by unmeasured comorbidities or subtle medication effects cannot be definitively ruled out.

Future investigations should prioritize prospective, randomized, double-blind, sham-controlled trials comparing standardized MAH protocols with oxygen-only or saline control groups in well-characterized MCI and cognitive frailty cohorts. Studies should incorporate (i) alternate parallel forms of cognitive and olfactory tests to minimize learning effects, (ii) extended follow-up periods (6–24 months) to assess durability and predict conversion to dementia, and (iii) comprehensive multimodal biomarker panels—including peripheral inflammatory and oxidative stress markers, endothelial function assays, structural/functional neuroimaging, and, where ethically and clinically appropriate, cerebrospinal fluid or plasma phosphorylated tau and amyloid-β—to elucidate mechanisms of action. Stratification by baseline olfactory status, MoCA Memory Index Score, and apolipoprotein E4 genotype may identify individuals most likely to benefit. Comparative effectiveness studies embedding MAH within multidomain lifestyle and cognitive rehabilitation programs are also needed to determine its incremental value over guideline-concordant non-pharmacological care.

### 4.5. Conclusions

This study suggests that a 5-week course of MAH in carefully selected individuals with MCI is associated with improved olfactory performance and enhanced cognitive function on sensitive instruments, with correlated changes indicating a shared and potentially modifiable pathophysiological substrate suggesting shared pathophysiological links between chemosensory and cognitive domains in MCI. These findings extend the established role of olfactory dysfunction as an early biomarker of neurodegeneration by introducing the hypothesis that short-term chemosensory performance in MCI may be amenable to change in association with systemic interventions; this hypothesis requires confirmation in sham-controlled randomized trials with mechanistic endpoints. Chemosensory impairments in MCI may be at least partially reversible through targeted redox and microvascular modulation. While confirmation in rigorously controlled trials is essential before clinical implementation, our data support further translational and clinical investigation of oxygen–ozone therapy as a biologically grounded, system-level intervention in the prodromal stages of cognitive decline.

## Figures and Tables

**Table 1:** Baseline socio-demographic characteristics of MCI and Control participants.

Variable	MCI (n = 81)	Control (n = 93)	Significance (*t*-Test, χ^2^)
Gender	male = 45 (55.5%) female = 35 (44.4%)	male = 51 (54.83%) female = 42 (45.16%)	χ^2^ = 0.01, *p* = 0.924
Education (years)	10.47 ± 2.08 [10.01, 10.93]	10.85 ± 2.20 [10.40, 11.30]	*p* > 0.05
BMI (Kg/m^2^)	27.66 ± 3.45 [26.90, 28.43]	27.24 ± 3.63 [26.49, 27.98]	*p* > 0.05
Age (years)	66.00 ± 7.99 [64.23, 67.77]	67.46 ± 8.63 [65.68, 69.23]	*p* > 0.05

Cells show mean ± SD [95% CI] for continuous variables. Gender is reported as n (%) for male and female. Two-tailed *t*-test for continuous variables (reported as *p* < 0.05 or *p* > 0.05); chi-square test for Gender (reported as χ^2^ and *p*-value).

**Table 2:** Within-patient and between-group effects of olfactory and cognitive testing MCI patients for pre-and post-MAH and control group participants.

	Pre (n = 81) Mean ± SD [95% C.I.]	Post (n = 81) Mean ± SD [95% C.I.]	Cohen *d* (Pre–Post)	Control (n = 93) Mean ± SD [95% C.I.]	Significance
***Olfactory Testing***					
**OT**	4.84 ± 2.72 [4.24, 5.45] **†**	5.77 ± 2.85 [5.13, 6.40] **‡**	0.33	6.68 ± 1.71 [6.33, 7.03]	F(2, 246) = 11.907, *p* < 0.05
**OD**	9.99 ± 1.15 [9.73, 10.24] **†**	10.40 ± 1.36 [10.09, 10.70] **‡**	0.33	11.92 ± 2.43 [11.42, 12.43]	F(2, 246) = 29.370, *p* < 0.05
**OI**	10.81 ± 3.49 [10.04, 11.59] **†**	11.89 ± 2.89 [11.25, 12.53]	0.34	12.73 ± 1.78 [12.37, 13.10]	F(2, 246) = 9.6599, *p* < 0.05
**TDI**	25.65 ± 5.24 [24.49, 26.80] *†	28.05 ± 5.11 [26.92, 29.18] **‡**	0.46	31.34 ± 2.74 [30.77, 31.90]	F(2, 246) = 33.871, *p* < 0.05
***Cognitive Testing***					
**MMSE**	24.46 ± 1.96 [24.02, 24.89] **†**	24.58 ± 1.97 [24.14, 25.02] **‡**	0.06	29.28 ± 0.90 [29.09, 29.47]	F(2, 246) = 230.28, *p* < 0.05
**Visuospatial**	4.22 ± 0.65 [4.08, 4.37] **†**	4.31 ± 0.65 [4.17, 4.45] **‡**	0.14	4.72 ± 0.45 [4.63, 4.81]	F(2, 246) = 19.7, *p* < 0.05
**Naming**	2.94 ± 0.24 [2.88, 2.99]	2.95 ± 0.21 [2.90, 3.00]	0.04	2.99 ± 0.10 [2.97, 3.01]	F(2, 246) = 1.584, *p* > 0.05
**Attention**	2.56 ± 0.63 [2.42, 2.70] **†**	2.63 ± 0.60 [2.50, 2.76]	0.11	2.80 ± 0.41 [2.71, 2.88]	F(2, 246) = 4.5788, *p* < 0.05
**Calculation**	2.58 ± 0.52 [2.47, 2.70] †	2.67 ± 0.52 [2.55, 2.78] ‡	0.17	2.99 ± 0.10 [2.97, 3.01]	F(2, 246) = 21.77, *p* < 0.05
**Language Repetition**	1.56 ± 0.57 [1.43, 1.68] *†	1.77 ± 0.48 [1.66, 1.87]	0.40	1.84 ± 0.37 [1.76, 1.91]	F(2, 246) = 8.551, *p* < 0.05
**Language Fluency**	0.72 ± 0.45 [0.62, 0.82] **†**	0.78 ± 0.63 [0.64, 0.92]	0.11	0.88 ± 0.32 [0.81, 0.95]	F(2, 246) = 3.216, *p* < 0.05
**Abstraction**	1.60 ± 0.49 [1.50, 1.71] **†**	1.77 ± 0.43 [1.67, 1.86]	0.37	1.89 ± 0.31 [1.83, 1.96]	F(2, 246) = 9.264, *p* < 0.05
**Delayed Recall**	2.01 ± 1.25 [1.74, 2.29] **†**	2.33 ± 1.18 [2.07, 2.59] **‡**	0.26	3.72 ± 0.94 [3.53, 3.91]	F(2, 247) = 55.824, *p* < 0.05
**Orientation**	5.53 ± 0.63 [5.39, 5.67] **†**	5.62 ± 0.57 [5.50, 5.76] **‡**	0.15	6.00 ± 0.00 [6.00, 6.00]	F(2, 246) = 20.861, *p* < 0.05
**MoCA**	23.72 ± 1.68 [23.35, 24.09] *†	24.88 ± 1.84 [24.47, 25.28] **‡**	0.66	27.83 ± 1.15 [27.59, 28.06]	F(2, 247) = 152.91, *p* < 0.05

Cells show mean ± SD [95% Confidence Interval, C.I.]. * Post hoc significant within-subjects comparison in MCI patients before and after MAH protocol; † post hoc significant comparison between pre-MAH protocol in MCI patients and control group participants; ‡ post hoc significant comparison between post-MAH protocol in MCI patients and control group. Main exact *p*-values of post hoc comparisons are given in the text. OT, Odor Identification; OD, Odor Discrimination; OI, Odor Identification; TDI, composite score of OT, OD and OI; MMSE, Mini-Mental State Examination; MoCA, Montreal Cognitive Assessment; SD, Standard Deviation.

## Data Availability

The data that support the findings of this study are available from the corresponding author, upon reasonable request.
